# Letter to the Editor: Duration of Workplace Noise Exposure and Blood Pressure Among Rural Adult Weavers: A Cross‐Sectional Study

**DOI:** 10.1002/hsr2.73229

**Published:** 2026-09-10

**Authors:** Khadija Fayyaz, Aiman Faiz

**Affiliations:** ^1^ Karachi Metropolitan University Karachi Pakistan

Dear Editor,

We read with great interest the article published by Prince et al. [1]. Although the study provides insightful information about occupational health risks among power loom workers, there are several methodological issues that could affect how the results are interpreted. We respectfully like to bring out a few methodological issues that need more discussion.

First, the authors contributed to body mass index, age, and smoking, but they failed to include heat stress and shift work—two critical occupational hazards. Bangladeshi power loom industries are renowned for excessive ambient heat, poor ventilation, and exhausting night shifts that disrupt circadian rhythms and increase blood pressure. In a recent study, heat stress has been associated with higher ambulatory blood pressure, particularly during vigorous physical activity or heat exposure [2]. Second, a significant methodological flaw of this study is the absence of a comparison or control group. Because all individuals were selected from high‐noise power loom job settings, there was no group with less noise exposure to compare the prevalence of hypertension. Therefore, the question of whether the high blood pressure levels are the direct result of the occupational noise exposure or other common features of the rural industrial workers such as long working hours, poor nutritional habits, occupational tension, and socio‐economic stress, remains uncertain. Prior studies evaluated the relationships of systolic blood pressure (SBP) and diastolic blood pressure (DBP) with occupational noise exposure in 1390 occupational noise‐exposed workers (exposed group) and 1399 non‐noise‐exposed control subjects (control group) [3]. In contrast, the present study focused solely on highly exposed workers, limiting the attribution of hypertension exclusively to occupational noise. Future studies should include appropriately matched control populations to improve internal validity and allow more definitive analysis of the effects of occupational noise on those of underlying socioeconomic or behavioral factors associated with hypertension.

Furthermore, the gender imbalance in this study is substantial: 94.1% male vs. only 17 female participants. This imbalance makes the gender comparison difficult to interpret. It is not possible to have reliable estimates with a sample size of only 17 women. Running linear models or group comparisons on such a small subgroup is likely to result in unstable results. In addition, recent research shows that when data are nonlinear and heterogeneous, a linear model may capture part of the pattern but fail to represent the rest [4]. Given these limitations, the authors' finding that females had higher mean systolic blood pressure (SBP) and diastolic blood pressure (DBP) should be treated with caution. The present sample does not support a strong conclusion on this point.

We appreciate the author's valuable contribution to this important area of occupational health research and hope these comments will help strengthen future studies and ongoing scientific discussion.

## Author Contributions

**Khadija Fayyaz:** conceptualization, writing – original draft, supervision. **Aiman Faiz:** supervision, writing – review and editing.

## Funding

The authors have nothing to report.

## Ethics Statement

The authors have nothing to report.

## Conflicts of Interest

The authors declare no conflicts of interest.

## Data Availability

Data sharing is not applicable to this article, as no new data were created or analyzed in this study.
